# Nitro-Muriatic Acid as a Remedial Agent

**Published:** 1840

**Authors:** 


### NITRO-MURIATIC ACID AS A REMIDIAL AGENT.

Dr. Mettauer has some valuable observations in the American Journal of the Medical Sciences for February, on the use of the nitro-muriatic acid. He has used it with the happiest effects in cases of obstinate constipation, puerperal mania, torpor of the liver, dyspepsia, uterine affections of a sub-inflammatory character, some of the forms of external scrofula, and nearly every variety of pseudo-syphilis. He remarks:

"From the prompt and decided action of this remedy upon the biliary secernents, when endermically applied in all the cases in which we have used it, it presents itself as a valuable remedy in the treatment of that scourge, Asiatic cholera; and should we ever have an opportunity of treating a case of that appalling disease, it shall constitute one of our chief remedies. In tnis disease it may be used both as a certain and prompt promoter of the biliary, and general secretory operations; and as a powerful revellent, and counter-irritant, in aiding the centrifugal tendencies to the skin, two of the most important therapeutic designs in treating cholera. It may also be beneficially employed in cases of irritability of the stomach demanding cathartics.

In the first case which has been detailed, we believe no other remedy would have arrested the course of the disease so promptly and effectually as the acid mixture; and from the numerous and satisfactory trials of other remedies unsuccessfully used, we are forced to the conclusion that no other agent possessed the therapeutic properties adapting a remedy to the cure of such a disease as this case must have been.

The case of puerperal mania furnishes evidence equally satisfactory, in support of the therapeutic powers of the acid mixture, when employed as an epidermic remedy, as no other agent was used at the time, nor had been employed for more than ten days before it was resorted to. In this case we are disposed to believe, if the acid mixture had not been employed externally, that the disease would have terminated fatally, as it was next to an impossibility to introduce efficient remedies in any other mode, and the patient was daily and rapidly declining, when it was commenced with, and must very soon have succumbed under the afflictive disease.

In the last case, particularly detailed, it will be conceded that this valuable remedy was equally efficient in arresting the tendency of the bowels to troublesome constipation, and in imparting to the digestive system a condition more favorable for a heathful exercise of its functions.

We believe the acid mixture is especially adapted to the treatment of morbid states of the human body, based in chronic inflammation, or engorgements of the capillary and parenchymatous structures of an indolent nature, either as the primary pathological condition of these structures, or the consequence of inflammation. In this view it is assimilated in its remedial action to mercury, to which agent we believe it bears a very striking resemblance in many of its therapeutic powers, especially those from which a deobstruent operation may be supposed to result. In the cases which have been detailed, as well as the diseases referred to, in which the remedy has been found decidedly beneficial, one or the other of these conditions must have obtained in the organs chiefly affected, or the pathological state from which the functional disturbances followed, constituting the external morbid phenomena of the disease present; the almost entire absence of constitutional fever, and the ordinary concomitants of a state of active phlogosis, in the diseases treated by us, as well as the cases reported in this communication, in which the acid mixture was chiefly beneficial, render it strongly presumable, that such was the character of their pathological basis in the structure at the time the remedy was employed.

In diseases of decided inflammatory character, the acid mixture is entirely inapplicable; and, as far as our experience enables us to decide, becomes so by reason of its tendency to irritate the constitution through those qualities which directly exalt the action of the economy unduly, so as to superinduce a state of hypersthenia. We have never found any but injurious effects to follow its action in every form of acute disease in which we have employed the remedy, and for a number of years have confined its use exclusively to chronic affections with very slight febrile disturbance of the general system.

Whether employed internally or externally, the remedial effects of the acid mixture are the same. Occasionally it acts with undue violence on the liver and bowels producing hypercatharsis; or even a condition may follow closely verging on dysentery, as it sometimes elicits sanguineous dejections, attended with tenesmus of most painful character. But, generally, its action is moderate and equable, and the remote textures, especially those forming the capillary and parenchymatous structures, as they constitute the glandular and other secsteting organs, are chiefly obnoxious to its operation. Upon the glandular system, as well as the extended secerning mucous and serous surfaces, it acts with peculiar effect as a promoter of secretion and absorption. It is very certain that this mixture increases the salival flow; but its action as a sialagogue differs from that of mercury in not producing tumefaction of the gums, but only slight tenderness of them, beyond which it can seldom be increased. The breath is never contaminated by it, unless the acid is permitted to act on the teeth; and to prevent this accident, it should always be imbibed through a tube of some indestructible material introduced fairly into the fauces.

From the experiments of Dr. Scott, and others, as well as from our individual experiments with the nitro-muriatic mixture, and a solution of chlorine in water, we are disposed to refer the action of the compound in every case to the presence of chlorine, and believe, with Dr. Scott, that a solution of this elementary substance in water will answer as well as the nitro-muriatic mixture as a remedial agent.

